# Large bronchogenic cyst of stomach, a case report of a rare congenital anomaly as an incidental finding during bariatric surgery

**DOI:** 10.1016/j.ijscr.2025.111658

**Published:** 2025-07-09

**Authors:** Mavlonbeg Karimov, Neda Haghighat, Tahere Mohseniyan Sisakht, Nader Moeinvaziri

**Affiliations:** a Laparoscopy Research Center, Shiraz University of Medical Sciences, Shiraz, Iran

**Keywords:** Bariatric surgery, Obesity, Bronchogenic cyst, Stomach, Case report

## Abstract

**Introduction and importance:**

Bronchogenic cysts are rare congenital anomalies of the foregut that occur as a result of abnormal budding during early embryogenesis.

**Case presentation:**

Here, we present a 21-year-old woman with a BMI of 47 who was candidate for one anastomosis gastric bypass surgery. During the operation we found two large cystic structures originating from gastric fundus and gastroesophageal junction as an incidental finding. So plan of surgery shifted to the sleeve gastrectomy as we had to remove the cysts while operating. Histological examination confirmed the bronchogenic origin of the cyst, with IHC confirmation.

**Clinical discussion:**

Although bronchial cysts are often asymptomatic, just like our reported case, the clinical manifestations of abdominal bronchogenic cysts depend on their size and location. If the cyst enlarges, it may compress adjacent organs, leading to abdominal pain, difficulty swallowing (dysphagia), or even bowel obstruction.

**Conclusion:**

Since the diagnosis of bronchogenic cysts is challenging preoperatively in individuals with obesity, every bariatric surgeon should have sufficient mental preparation to adopt the appropriate surgical method when faced with this case.

## Highlights

• We present a rare case of intraoperative detected bronchogenic cysts of the stomach in the gastric fundus and gastroesophageal junction in a patient who underwent bariatric surgery.
• Although bronchial cysts are often asymptomatic, their enlargement can cause compression of adjacent organs, leading to symptoms such as cough, shortness of breath, dysphagia, or recurrent infections.
• Bariatric surgeons should consider bronchogenic cysts in the differential diagnosis of gastric cystic lesions, as early surgical intervention ensures excellent prognosis and prevents potential complications

## 1 Introduction

Bronchogenic cysts are rare congenital anomalies of the foregut that occur as a result of abnormal budding during early embryogenesis [1]. They are most commonly located in the mediastinum or lungs, but very rarely can be found in the abdominal cavity, including the stomach [2]. We present a case of intraoperative detection of two bronchogenic cysts in the region of the gastric fundus and gastroesophageal junction in a patient who underwent bariatric surgery. The cysts were an incidental finding, since the patient had no specific complaints. Histological examination confirmed the bronchogenic origin of the cyst, which was further confirmed by immunohistochemically (IHC) analysis. According to the literature, the corresponding case was reported in 1956; as of 2019, a total of 37 cases of gastric bronchogenic cysts were identified in the Medline and PubMed medical databases, most of which were detected incidentally or manifested by episodic epigastric pain [3]. The diagnosis of bronchogenic cysts is challenging because they are rarely recognized preoperatively and may be mistaken for stromal tumors. Imaging with CT or MRI plays a key role in the detection of these cysts; however, a definitive diagnosis is established only after histological analysis. Symptomatic cysts may cause compression symptoms, infections, or, rarely, malignancy, making surgical removal the preferred treatment option. In our case, the cysts were resected uneventfully and the patient had no recurrence in the postoperative period. Given the rarity of this pathology, including it in the differential diagnosis of cystic lesions in the abdominal cavity may improve the diagnostic accuracy. Surgical treatment is recommended even in asymptomatic cases to prevent potential complications. To the best of our knowledge, this is first reported case of incidentally founded stomach bronchogenic cyst while performing bariatric surgery. This case report has been reported in line with the SCARE criteria [4].

## 2 Case presentation

### 2.1 Patient information

A 21-year-old woman with a body mass index (BMI) of 47 presented to the clinic seeking medical assistance for severe obesity. She had no gastrointestinal complaints, no harmful habits, and no significant family or personal medical history. Preoperative evaluation revealed a binge eating disorder, and one-anastomosis gastric bypass (OAGB) was recommended. Laboratory tests and endoscopy were unremarkable, while ultrasonography showed grade I–II hepatic steatosis and two simple cysts in the left upper quadrant (33 × 30 mm and 62 × 43 mm), suspected to be renal cysts. Since there were no clinical or laboratory abnormalities, further imaging with contrast-enhanced CT was not indicated.

### 2.2 Operation findings

**Fig. 1:**
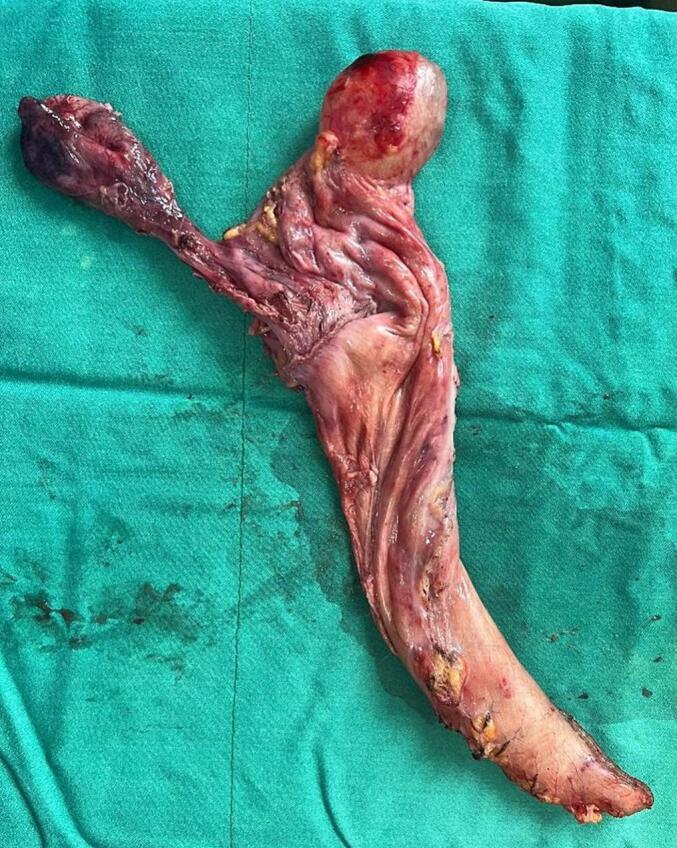
Resected stomach along with two bronchogenic cyst.

**Fig. 2:**
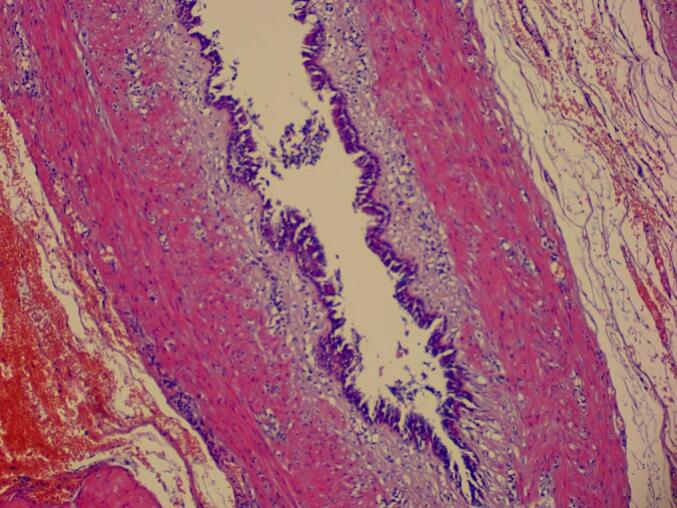
Pathological result in favour of bronchogenic cyst.

**Fig. 3:**
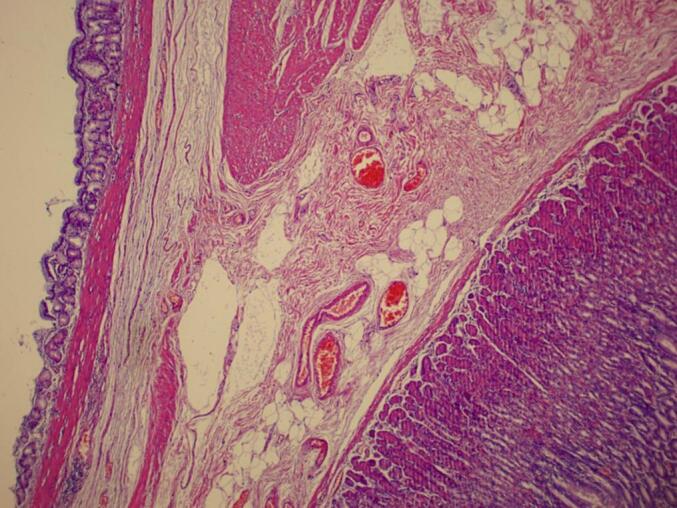
Pathological result in favour of bronchogenic cyst.

**Fig. 4:**
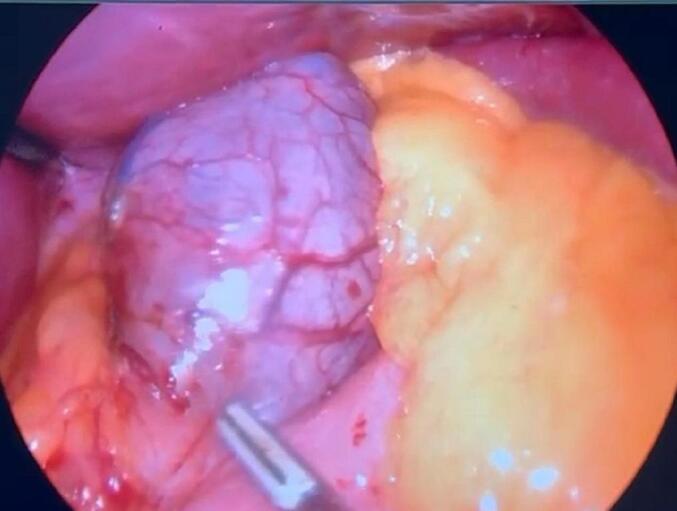
Intraoperative view of bronchogenic cyst of stomach.

During the operation, a large cystic lesion was discovered in the upper part of the stomach near the splenic hilum. It was completely mobilized and identified as a large simple gastric cyst originating from proximal part of gastric fundus near the GE junction. So we shifted to the alternative plan of sleeve surgery as we had to remove the cyst while operating. After complete release of omentum from the greater curvature, another smaller cyst was found just four cm below the previous cyst arising from the fundus (Fig. 1, Fig. 4). Sleeve gastrectomy was performed on a 36 Fr size bougie using five 60-mm purple Endo-GIA stapler, with reinforcement using Prolene 2.0. leak test was negative, hemostasis was secured, and a Jackson-Pratt drain was placed through the epigastric port. The resected portion of the stomach along with its cysts was removed through the left upper quadrant port, and the fascial defect was closed with Vicryl 1. Macroscopic examination of the resected specimen revealed an irregular 5×4 cm cyst on the gastric fundus, 1.5 cm from the angle of His, and another smaller cyst four cm distal to the previous cyst containing yellowish serous fluid. Histological analysis confirmed congenital intra-abdominal benign bronchogenic cyst. No pathological changes were detected in the gastric mucosa, muscularis propria, or serosa (Fig. 2, Fig. 3). Immunohistochemical analysis confirmed the presence of respiratory ciliated pseudostratified epithelium. This case highlights the incidental discovery of a bronchogenic stomach cyst in a patient undergoing bariatric surgery, emphasizing the importance of thorough pre- and intraoperative evaluation in such procedures.

### 2.3 Follow up and hospital course

The patient was started on fluids the day after surgery, which were well tolerated. With stable conditions and normal laboratory tests, the patient was discharged two days after surgery with appropriate dietary recommendations. One week postoperatively, a follow-up visit was conducted, during which the drain was removed and necessary supplements were prescribed. The last follow-up visit was approximately two months after the surgery, at which time an ultrasound was performed, showing normal findings with no evidence of cysts and the patient had lost approximately 16 kg in weight.

## 3 Discussion

A bronchial cyst is a congenital anomaly that arises due to deviations in the development of the foregut during early embryogenesis. Normally, between the 3rd and 5th weeks of intrauterine development, the ventral diverticulum forms the tracheobronchial tree. However, if this process is disrupted, isolated cystic structures may develop, unconnected to the airways [3]. The location of the cyst depends on the timing of its formation. If the anomaly occurs at an early stage, the cyst is usually found in the mediastinum, whereas later disruptions lead to cyst formation within the lung parenchyma. In rare cases, such formations can be found in the stomach, diaphragm, or even the retroperitoneal space [5]. Structurally, bronchial cysts have walls containing elements characteristic of the respiratory system, including ciliated epithelium, smooth muscle, hyaline cartilage, and glandular tissue. These features help distinguish them from other cystic formations [6]. Although bronchial cysts are often asymptomatic, their enlargement can cause compression of adjacent organs, leading to symptoms such as cough, shortness of breath, dysphagia, or recurrent infections. Due to the risk of complications, including infection and potential malignancy, surgical removal is considered the optimal treatment method [7]. Our reported case did not have any dysphagia or abnormal complaint prior to operation. Preoperative workups such as endoscopy were completely normal but sonography reported large simple cyst in the left upper part of abdomen (simple renal cyst) which probably was a misdiagnosis. The mandatory preoperative evaluations include endoscopy and abdominal and pelvic ultrasound. If any suspicious findings are detected, a CT scan is definitely requested for more accurate diagnosis. Considering that our patient had no specific complaints or symptoms and only a simple renal cyst was reported on the preoperative ultrasound, we did not see the need for further diagnostic evaluations or imaging. However, it should be noted that one of the diagnostic limitations of preoperative assessment protocols for patients with morbid obesity is performing a CT scan, which is only done in very limited centers in our city due to the risk of damage to the equipment. Prevalence ranges from 1 in 42,000 to 1 in 68,000 individuals, according to data from surgical interventions and autopsy studies [8] Due to the rarity of this pathology and its often asymptomatic course, precise epidemiological data on the prevalence of abdominal bronchogenic cysts remain limited [9,10]. However, in recent years, an increasing number of retroperitoneal extra visceral cysts have been diagnosed, which may be attributed to advancements in imaging techniques and growing awareness of this anomaly [11]. The clinical manifestations of abdominal bronchogenic cysts depend on their size and location. If the cyst enlarges, it may compress adjacent organs, leading to abdominal pain, difficulty swallowing (dysphagia), or even bowel obstruction. Diagnostic methods include imaging techniques such as ultrasound (US), computed tomography (CT), and magnetic resonance imaging (MRI), which help determine the exact location and size of the cyst [9].Surgical removal remains the primary treatment method, as it prevents complications such as infection and, in rare cases, malignancy [10]. The prognosis after timely surgical intervention is generally favorable, as complete cyst excision eliminates the risk of recurrence and potential complications [11]. According to our center's protocol, patients undergoing bariatric surgery are subjected to comprehensive check-up tests every three months in the first year of post-operative period. Therefore, since at the time of writing this article only about two months have passed from the patient's surgery, no laboratory tests were available for reporting. During the two months following the surgery, the patient experienced approximately 16 kg weight loss without any significant problem or complaint. Additionally, no specific findings were reported in the ultrasound performed at the two-month mark. We believe that this paper is the first reported case of large stomach bronchogenic cyst arising from fundus which led to shifting the plan of bariatric surgery.

## 4 Conclusion

This case underscores the necessity of comprehensive intraoperative evaluation during bariatric surgery, especially in obese patients where preoperative imaging may not fully reveal abdominal anomalies. The unexpected identification of bronchogenic cysts may require a shift in the surgical plan, demonstrating the importance of adaptability in surgical decision-making. Bariatric surgeons should consider bronchogenic cysts in the differential diagnosis of gastric cystic lesions, as early surgical intervention ensures excellent prognosis and prevents potential complications.

## CRediT authorship contribution statement

Writing the paper and study concept: Mavlonbeg Karimov. Data collection and study concept: Neda Haghighat and Tahere Mohseniyan Sisakht. Supervision: Nader Moeinvaziri.

## Consent

Written informed consent was obtained from patient and her family.

## Ethical approval

Ethical approval was waived by the Research Ethics Committee. Case reports involving a single patient encountered during routine clinical care are exempt from ethics approval at our institution.

## Guarantor

Dr Nader Moeinvaziri.

## Research registration number

None.

## Funding

This case report received no specific grant from any funding agency in the public, commercial, or not-for-profit sectors.

## Declaration of competing interest

There is no conflict of interest in this study.
